# EHD‐LIFT: Electrohydrodynamic Laser Induced Forward Transfer

**DOI:** 10.1002/advs.78600

**Published:** 2026-10-31

**Authors:** Ankit Das, Jordi Torres‐Durall, J. Marcos Fernández‐Pradas, Chien‐Fang Ding, Martí Duocastella, Craig B. Arnold

**Affiliations:** ^1^ Department of Mechanical and Aerospace Engineering Princeton University Princeton USA; ^2^ Princeton Materials Institute Princeton University Princeton USA; ^3^ Department of Applied Physics Universitat De Barcelona Barcelona Spain; ^4^ Institut De Nanociència i Nanotecnologia (IN^2^UB) Universitat De Barcelona Barcelona Spain; ^5^ Department of Biomechatronics Engineering National Taiwan University Taipei Taiwan

**Keywords:** electric fields, electrohydrodynamic forces, flow‐focusing, jetting dynamics, laser induced forward transfer

## Abstract

Adding new degrees of control to established manufacturing techniques can significantly expand their functionality and unlock new application spaces. In laser‐induced forward transfer (LIFT) of liquids, jetting characteristics dictate the transfer features, essential for micro and sub‐microscale patterning. However, a simple strategy for dynamic tunability of the jet beyond conventional laser parameters is still lacking. Here, we introduce an electrohydrodynamic method to control the donor interfacial curvature, enabling precise tuning of the jetting dynamics through the flow focusing effect. As a result, narrower jets could be achieved at energies 66% lower than in conventional LIFT, leading to a 97% reduction in the minimal printed droplet radius. By introducing an additional degree of control independent of laser parameters, this method expands the operational capabilities of LIFT and provides a promising pathway for high‐resolution printing of functional materials, with potential applications in microelectronics, biomedical systems, and advanced engineering technologies.

## Graphical abstract

Electrohydrodynamic laser‐induced forward transfer (EHD‐LIFT) introduces electric field‐assisted control to LIFT. Through laser‐patterning a conductive absorber layer, local curvatures are induced in the donor film, enabling flow‐focusing for transfer of smaller droplets at lower laser energies. Through this additional degree of process control, EHD‐LIFT expands the tunability of LIFT and enables higher‐resolution printing across a broad range of functional materials.

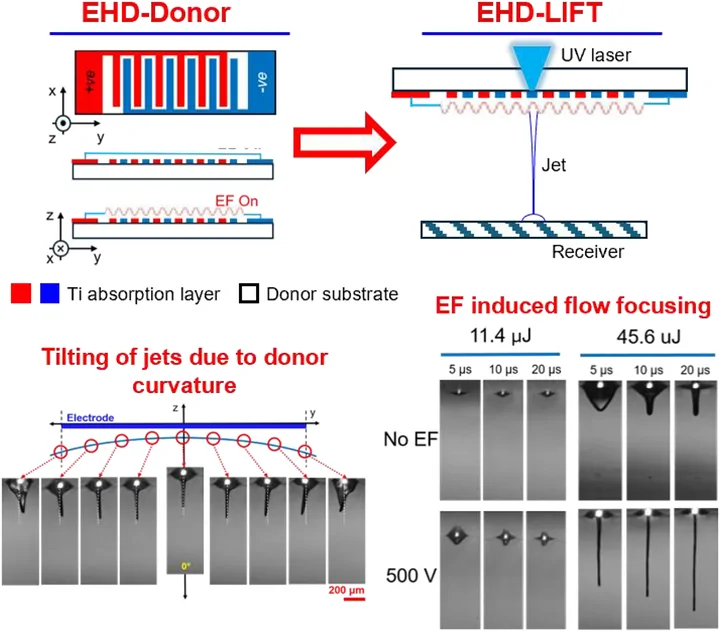

## 1 Introduction

Laser‐induced forward transfer (LIFT) has been an exceptionally versatile technique for additive manufacturing of solid [1] and liquid materials [2] for a wide range of applications [3, 4], often from a donor material to a receiver substrate using a pulsed laser [5]. In particular, liquid donors have enabled functional patterning for fields such as optics [6], electronic/energy devices [7, 8], tissue engineering [9, 10], and sensors [11]. Owing to its non‐contact and high resolution nature, LIFT has become a powerful approach for manufacturing structures at micrometer length scales. The geometrical characteristics of the transferred material are governed by several process parameters, which influence the underlying transfer mechanism. For liquid donors, the jetting dynamics during transfer play a dominant role in determining the final feature size and morphology. Consequently, strategies for modulating jet formation remain an important focus for advancing the capabilities of the LIFT process.

Conventionally, control over jetting dynamics is achieved by tuning LIFT processing parameters such as laser pulse energy, donor thickness, and laser spot size. Often these parameters are optimized for controlling transferred volumes, from picoliter to the femtoliter range, depending on the donor material properties and the specific process configuration. Introducing an intermediate layer in between the donor substrate and the donor material can assist the LIFT process. For example, this intermediate layer can be a metallic absorber layer or a polymer layer as used in blister‐actuated LIFT (BA‐LIFT) [12]. Beyond parameter optimization, fluid dynamics based approaches have also been explored to alter jet characteristics through mechanisms such as flow focusing [13]. Concave surface geometries are particularly effective in promoting flow focusing and facilitating the formation of slender jets [14, 15]. Previous studies have demonstrated this concept using acoustic excitation (∼100 Hz) to generate Faraday waves that create transient concavities on the donor surface [14]. Alternatively, micro‐fabricated holes have been employed to confine concave menisci that assist jet focusing during transfer [15]. While these approaches demonstrate the potential of altering geometry‐assisted jet, easy control in the tunability of the interfacial curvature is missing and they often require specialized equipment or complex fabrication steps, limiting their accessibility and scalability. Simply put, the development of a simpler and more universally applicable method for delicate control over the interfacial donor curvature in LIFT remains desirable.

External electric fields (EFs) offer a promising route for actively manipulating liquid interfaces. EFs can deform fluid films through mechanisms such as electrowetting and electrohydrodynamics (EHD), enabling controlled modulation of surface profiles [16, 17]. Depending on the electrode configuration, localized convex and concave deformations can be induced in liquid films in either: (i) horizontal [18, 19], or (ii) vertical [20] EF orientations. Horizontal configurations typically employ interdigitated electrodes patterned on the same substrate that supports the liquid film [21, 22], whereas vertical configurations involve electrodes placed on parallel substrates with the liquid film located on one of them [20]. This positions EFs as a promising candidate for active control of the donor interface in LIFT and extend its operational limits.

In this work, we demonstrate an EHD assisted LIFT approach for transferring a viscous liquid donor (∼300 cP) using EF induced flow focusing. Unlike conventional EHD jet printing, in which EF induced stresses directly deform the liquid meniscus and drive jets through the formation of a Taylor cone [23], EHD‐LIFT uses the EF primarily to precondition the donor interface by generating localized concave curvature. The subsequent jet formation and material transfer are driven by the laser induced bubble dynamics, while the imposed curvature promotes flow focusing and provides an additional means of controlling jet evolution independently of the laser parameters. Moreover, the nozzle free and inessential requirement of Taylor cone formation in LIFT makes EHD‐LIFT a simpler process [24]. A DC EF is applied through interdigitated titanium electrodes in a horizontal configuration, which simultaneously serve as the absorbing layer for the LIFT process. The applied EF induces localized surface deformations that generate concave regions at the donor interface, which are subsequently used as irradiation sites for initiating transfer. This field‐assisted donor curvature promotes flow focusing during jet formation, leading to reduced pulse energy requirements and smaller transferred volumes. Although gravity does not affect the LIFT process in itself, it makes handling and stabilization of liquid donors difficult. The strong EHD forces enable stable donor configurations independent of gravitational orientation, expanding the operational flexibility of the process. We characterize the effects of EFs on donor curvature and jetting dynamics, and analyze the resulting reductions in transfer feature size and threshold energy. As our results demonstrate, EHD‐LIFT enables reproducible control of the donor interface and jetting dynamics with ease, providing the additional degree of control that LIFT has long lacked.

## 2 Results

### 2.1 Electric Fields and Donor Interface

First, we investigated the effect that EFs have on the donor film. To this end, several interdigitated electrode configurations were examined, as illustrated in Figure 1a–c. In all cases, application of EFs deforms the liquid donor into a wave‐like profile, characterized by crests over the glass substrate and troughs located above the electrodes (+ve or ‐ve). This deformation varies with the geometry and applied voltage, as readily visualized using shadowgraphy (see Figure 1d–g). To quantitatively characterize this effect, confocal measurements were performed in the upright orientation (convexities opposing gravity). Under an applied voltage of 700 V, localized concavities are formed at the electrode locations, and their geometrical characteristics vary depending on both electrode configuration and donor thickness (Figure 1h,i). Furthermore, the concavities observed above the grounded electrodes (‐ve) differ from those above the live electrodes (+ve), as demonstrated for electrode configuration 3 in Figure 1i. Similar behavior is observed across all electrode geometries investigated. This asymmetry originates from the directional distribution of EF lines near the electrodes. Specifically, the field lines diverge outward from the live electrode and converge toward the grounded electrode, as illustrated in Figure S3.

**FIGURE 1:**
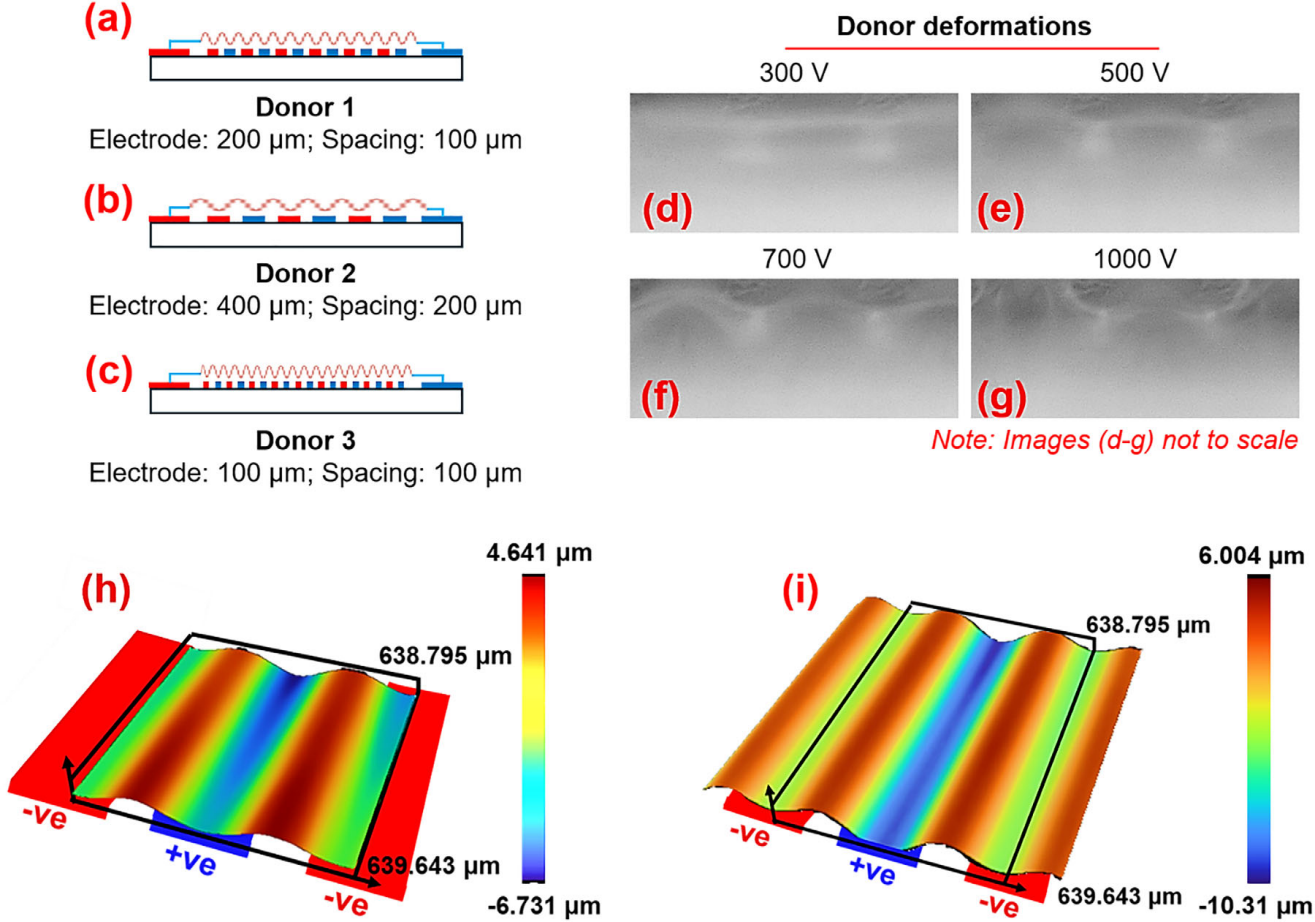
(a–c) Schematic illustration of the different donor electrode configurations in the upright orientation used with electric fields. (d–g) Shadowgraph images of the donor interface at different voltages for Donor 1 in the downward orientation. Confocal analysis showing the surface topography, (h) donor deformation on electrode 1 at 700 V and (i) donor deformation on electrode 3 at 700 V. Donor thickness: 170 µm.

Figure 2a presents the measured peak to valley lengths of the fluid interface as a function of applied voltage for different electrode configurations, revealing three distinct regimes. Up to 800 V, there is a stable operating regime where the interfacial deformation increases quadratically with the applied voltage. This quadratic dependence is expected because electrohydrodynamic forces (*F_ij_*) arise from Maxwell stresses within the liquid donor, which scale with the square of the electric field, $F_{ij}\propto E_{xy}^{2}$ [25, 26]. These localized forces generate the convexities and concavities observed at the interface. From approximately 800 to 1000 V, a transition regime appears which is a result of fluid instabilities/motion. Finally, an unstable regime is observed above ∼1000 V.

**FIGURE 2:**
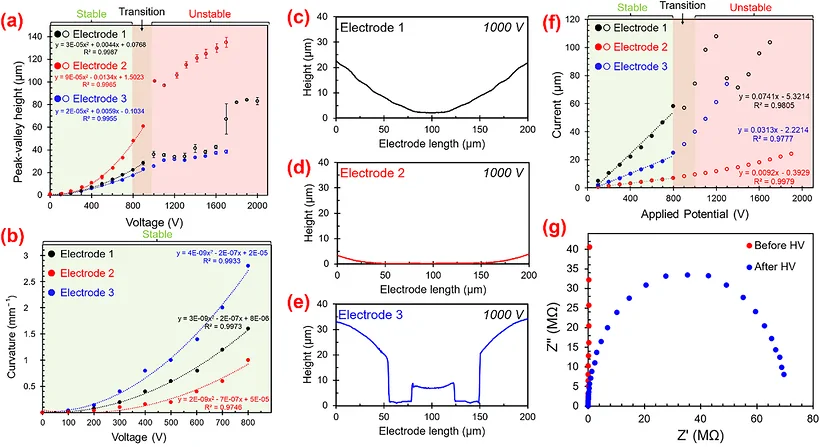
Experimental values of the (a) peak to valley height and (b) curvature of the donor interface at the ground (+ve) electrode. (c–e) Confocal profile of the donor interface for various electrode configuration (c) 1, (d) 2, and (e) 3 in the transition regime at 1000 V. (f) Current response during application of different EF magnitudes through the different electrode configurations. (g) Nyquist plots during EIS of the electrodes before and after experimental measurements through the unstable regime (at high voltages (HV) > 1000 V) shown in (f). Donor thickness: 170 µm.

An analysis of the curvature of the concavities above the grounded electrodes in the stable regime, which governs the flow focusing effect, is shown in Figure 2b. The measured curvature also follows a quadratic trend, directly reflecting the evolution of the peak to valley deformation. Subsequent sections reporting experimental results utilize the stable regime for demonstrating controllable and reproducible LIFT processing using EFs. Figure 2c–e shows the donor interfacial deformation above the electrode for different electrode configurations in the transition regime at 1000 V. In this regime, the concavities are not consistent in nature and are dependent on the electrode configuration. While Donor 1 with electrode configuration 1 shows concave interface (Figure 2c), Donor 2 shows a flat interface (Figure 2d). Donor 3, on the other hand, shows fluid instabilities with a central fluid channel with a concave interface (Figure 2e). The donor interfacial behavior in the transition regime is expected to change jetting behavior and is reported in the subsequent sections.

To further investigate the erratic behavior observed in the unstable regime (Figure 2a), additional measurements including current monitoring and electrochemical impedance spectroscopy (EIS) were performed. Amperometric measurements reveal two distinct current response regimes, suggesting a transition in interfacial behavior (Figure 2f). Moreover, EIS measurements conducted on pristine and electrically stressed electrodes show markedly different Nyquist responses for electrodes operated above the transition voltage (Figure 2g). These observations indicate that electrochemical interactions between the donor and electrodes along with strong fluid motions occur beyond the stable regime, leading to the unstable interfacial deformation. This renders operation above 1000 V unsuitable for reproducible LIFT processing. Video S1 shows fluid instabilities occurring at higher voltages.

### 2.2 Electric Fields in LIFT

#### 2.2.1 Effect of Pulse Energy on Jetting Dynamics

Pulse energy plays a critical role in the jetting behavior of liquid donors. A minimum pulse energy is required to initiate a jet capable of propagating without retracting under the restoring effects of surface tension and viscosity. Figure 3a presents time‐resolved images of the donor response under laser irradiation with and without the application of an EF. In the absence of an EF and at low pulse energies, a bubble is generated that subsequently collapses. Increasing pulse energy results in progressively larger bubble formation, and at 45.6 µJ, the onset of jet formation is observed. However, the resulting jet retracts back into the donor, indicating that the propulsive forces generated by the laser pulse are insufficient to overcome the restoring forces of the donor liquid. To generate a fully developed and propagating jet that, in the presence of a receiver substrate, would result in successful transfer, the pulse energy must be > 46 µJ (see Supplementary Information and Figure S1 for additional time‐resolved imaging experiments at higher pulse energies and longer process times).

**FIGURE 3:**
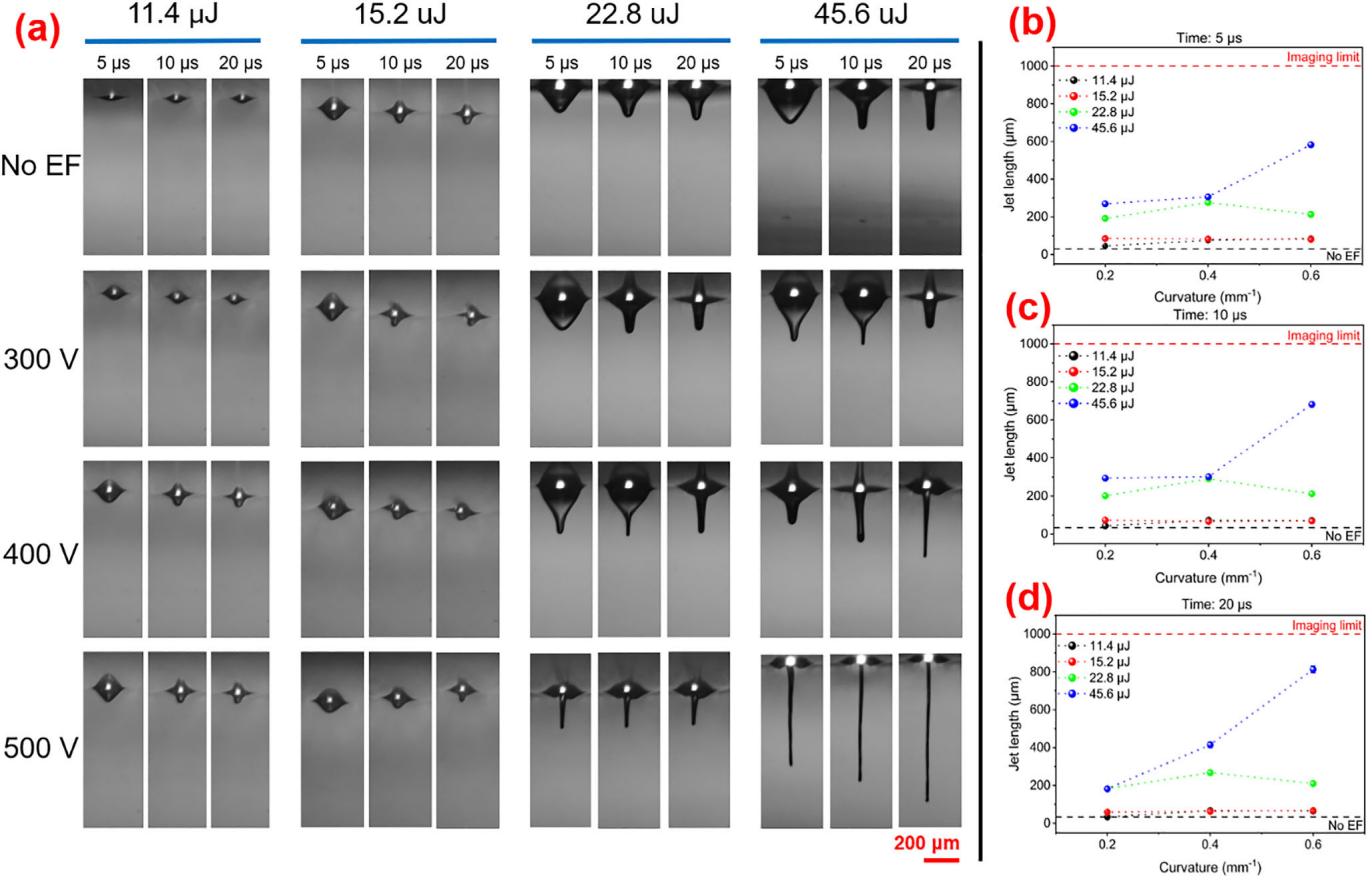
(a) Time‐resolved analysis of the jetting behavior with and without EFs. (b–d) Jet lengths measured under varying pulse energies and EFs: (b) 5, (c) 10, and (d) 20 µs.

The introduction of EFs promotes the formation of larger bubbles and protrusions at substantially lower pulse energies (∼22.8 µJ) (Figure 3a). More importantly, at applied voltages above 400 V, corresponding to a donor interfacial curvature of approximately ∼0.4 mm^−1^, stable jets are generated at 45.6 µJ that do not retract and are therefore suitable for transfer. Additionally, the application of EFs not only enables jetting at lower energies but also accelerates jet evolution. For example, comparison between 400 V (∼0.4 mm^−1^) and 500 V (∼0.6 mm^−1^) at the same pulse energy of 45.6 µJ demonstrates faster jet propagation at higher curvatures. This behavior, quantitatively analyzed in Figure 3b–d, originates from enhanced flow focusing induced by the locally concave donor interface. It should be noted that the data corresponding to 22.8 µJ represent a transition regime characterized by jet formation and retraction. This behavior is explored in greater detail in the following sections.

To further investigate the influence of pulse energy under EF‐assisted jetting conditions, pulse energies of 30.4, 38.0, and 45.6 µJ were examined at a constant voltage of 700 V, corresponding to a donor interfacial curvature of ∼1.2 mm^−1^, as shown in Figure 4a. Even when such high voltages are applied, the flow focusing effect alone is insufficient to sustain stable propagating long jets at pulse energies below 30.4 µJ. Note, though, that it is still possible to transfer material at these low energies by placing the receiver substrate within the jet length, a process known as the bridge transfer mechanism [27]. However, this type of transfer is not controllable, and thus, it is more reliable to utilize the jet propagation followed by the jet breakup mechanism [28]. Such long‐propagating jets are obtained for pulse energies exceeding 30.4 µJ (Figure 4b,c). Figure 4d shows the temporal evolution of these jets under different pulse energies. Consistent with prior works [15], an overall linear increase in jet length is observed with time; that is, the jets advance at constant speed. A slight deviation at early times is attributed to the transition between bubble collapse and jet formation.

**FIGURE 4:**
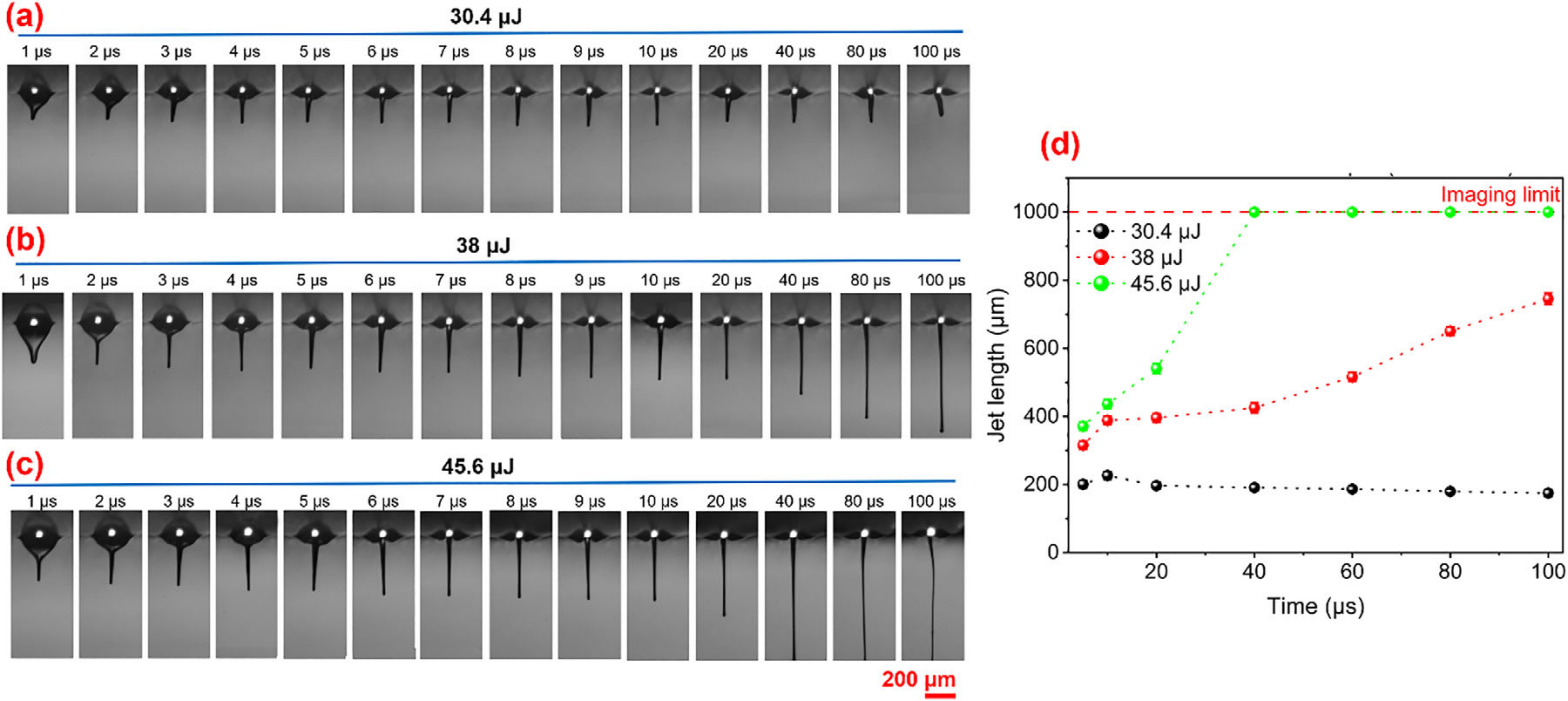
(a–c) Time‐resolved imaging and (d) quantitative analysis of the jetting behavior under a constant voltage of 700 V with a curvature of ∼1.72 mm^−1^ when irradiated with (a) 30.4, (b) 38, and (c) 45.6 µJ.

#### 2.2.2 Effect of EF Strength on Jetting Under Constant Pulse Energy

In typical LIFT processing, process parameters such as donor material properties and laser energy are used to optimize transfer. EFs offer an additional control mechanism for extended process tunability. Accordingly, Figure 5a presents time‐resolved images of the jetting behavior at different EF strengths while maintaining a fixed laser pulse energy of 55.1 µJ. In the absence of an applied EF, no stable elongated jet is formed at this pulse energy (Figure 5a). Figure S5a shows that the jet length decreases over time under no EF conditions due to jet retraction, whereas in the presence of EFs, elongated jets are produced with lengths exceeding 1000 µm (Figure 5a,b). Applied voltages of 300, 500, 700, and 1000 V correspond to donor interfacial curvatures of approximately ∼0.2, ∼0.6, ∼1.2, and ∼2.2 mm^−1^, respectively. As the voltage and corresponding curvature increase, stable elongated jets are generated rapidly, with fully developed jets appearing within 10 µs for voltages up to 1000 V (∼2.2 mm^−1^) as seen from Figure 5a,b. Quantitative measurements of jet lengths exceeding 1000 µm were constrained by the experimental setup and are therefore reported as saturated values in Figure 5b. Furthermore, the influence of donor curvature, and equivalently the applied voltage, on jet length appears to be approximately linear. This observation is consistent with previous studies [15], suggesting that the observed relationship is independent of the method used to generate the donor curvature (Figure 5c) and is likely associated with a flow focusing mechanism. Furthermore, the subsequent analysis of jet tilting provides additional experimental evidence supporting the role of flow focusing in EHD‐LIFT. However, the slope of this linear relationship changes at higher applied voltages (∼1000 V), indicating a possible synergistic interaction between the fluid dynamics governing the LIFT process and the electrohydrodynamic forces induced by the applied EF. Finally, the ability to control jet velocity through adjustment of the applied voltage, and consequently the donor interfacial curvature, is demonstrated in Figure 5d,e. A pronounced increase in jet velocity is observed with increasing voltage or curvature, followed by a tendency toward saturation at higher magnitudes, as shown in Figure 5d,e.

**FIGURE 5:**
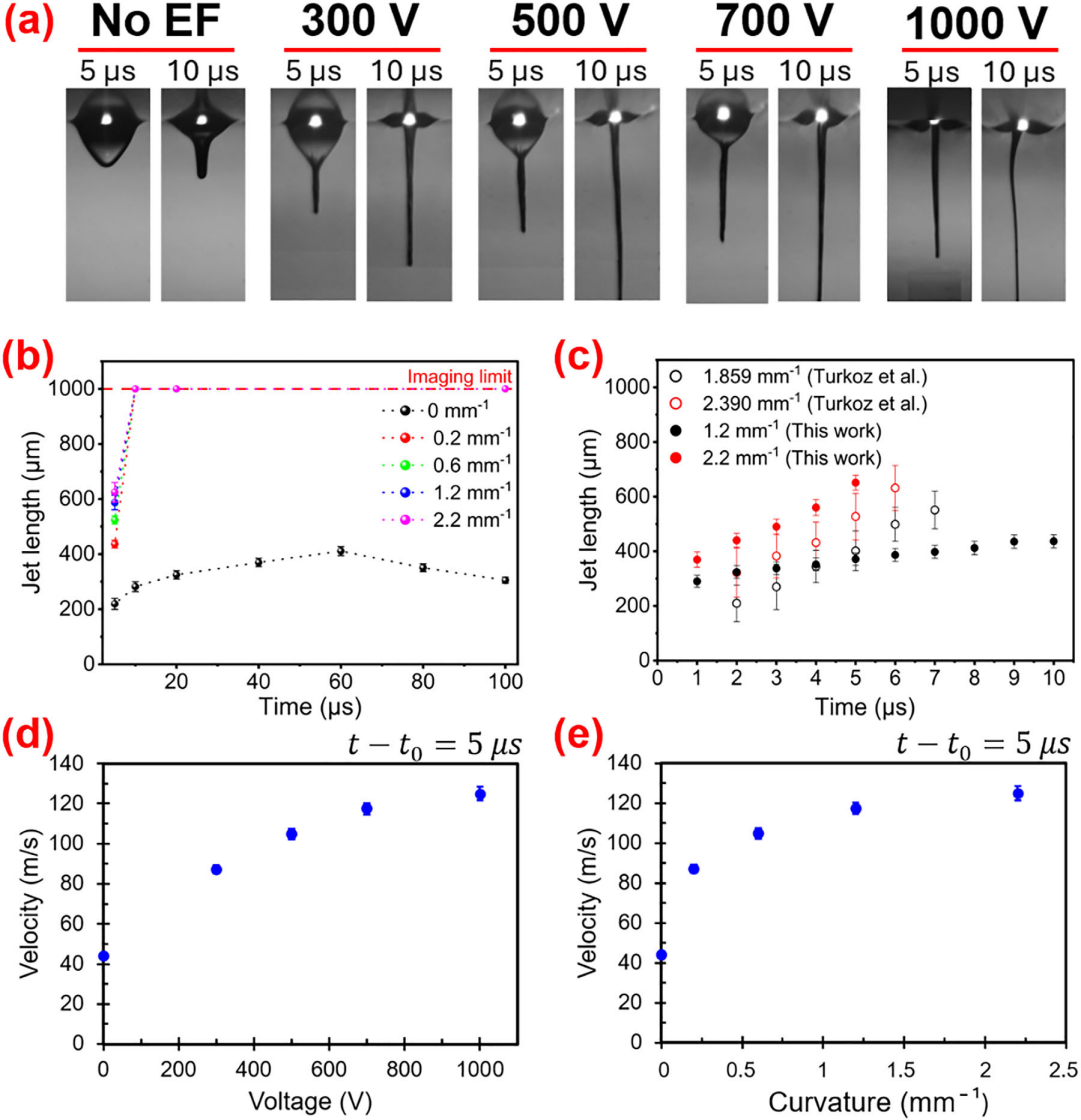
(a) Time‐resolved images of jetting behavior under fixed pulse energy (55.1 µJ) with (300, 500, 700, and 1000 V) and without EFs. (b) Quantitative illustration of jet length evolution under different curvatures. (c) Comparison in the effect of curvature produced by EFs and fabrication methods [15] on jet evolution (*Note*: Pulse energies: this work ∼45 µJ and previous work ∼6.5 µJ). Jet velocity as a function of (d) voltage and (e) curvature.

#### 2.2.3 Effect of Curvature on Jet Tilting

To further validate curvature as the driving mechanism for changes in jetting behavior, we investigated jet trajectory as a function of location relative to the local concavity. Based on the preceding characterizations, the minima of the concavities are located directly above the electrodes, as illustrated in Figure 6. Consequently, when the laser is irradiated away from the center of the concavity, the resulting jets exhibit measurable tilting. To investigate this effect, the 200 µm wide electrodes were divided into eight equal sections, and laser irradiation are performed at intervals of 25 µm across the electrode width, as shown in Figure 6a. In addition to enhanced flow focusing, systematic jet tilting is observed as a function of irradiation position relative to the donor curvature. Figure 6b shows a sinusoidal dependence of the jet tilting angle on electrode position. This behavior is expected from the geometry of the interface, as the jet is emitted along the local surface normal [29]. The local concavity curvature can be approximated as a sinusoidal surface and thus the normal is expected to vary similarly. For extended analysis, see Supporting Information.

**FIGURE 6:**
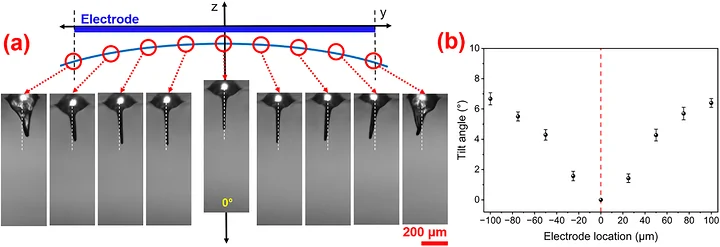
(a) Images and (b) graph showing tilting trends of jets at different locations of the curvature under a voltage of 500 V (∼0.6 mm^−1^), laser pulse energy of 45.6 µJ, and time stamp of 3 µs. *Note*: The location of irradiation is illustrated by red dashed circles at intervals of ∼25 µm. Width of the electrode: 200 µm; Gap: 100 µm.

## 3 Droplet Printing Under EHD‐LIFT

Our results demonstrate that manipulation of local donor curvature through externally applied EFs effectively modifies jetting dynamics during LIFT. As shown in Figure 7a, optimized EF conditions of ∼800 V, corresponding to a curvature of ∼2.36 mm^−1^, enable the formation of thin jets at substantially reduced pulse energies compared to conventional LIFT. Under these conditions, EF results in a significant extension of the operational range for droplet transfer. The range of droplet radii and corresponding volume is also broadened (Figure 7b,c). Likewise, Figure 7b,c reports all laser and EF parameters leading to successful material transfers. Under conventional LIFT conditions, a clear transfer threshold of approximately 44–46 µJ (leading to a droplet radius of ∼30.4 µm) is observed, below which no transfer occurs. In contrast, under an applied EF of 800 V, successful transfer with droplet radii as small as 905.5 ± 100 nm is achieved at pulse energies of ∼18 µJ. This drastic reduction in droplet size can be observed in Figure 7d–g. These results demonstrate that EHD‐LIFT enables both significantly smaller feature sizes (approximately 97.35% reduction in radii and 5 orders of magnitude reduction in volume) and substantially lower pulse energy requirements (approximately 60.41% reduction) compared with conventional LIFT Figure 7h,i. The presented results, were extracted from droplet arrays that were printed to the corresponding process parameters. Figure S12 shows droplet array results which were quantitatively analyzed to establish the repeatability and consistency of the results (Table S1). At energies close to the threshold, successful prints can be obtained, however, some limitations exist in terms of repeatability, and is believed to arise from fluctuations in the laser energy. Therefore, operation at energies higher than the threshold is encouraged (Figure S12). For droplet arrays printed using the EFs assisted curvature, successful and reproducible transfer can be made.

**FIGURE 7:**
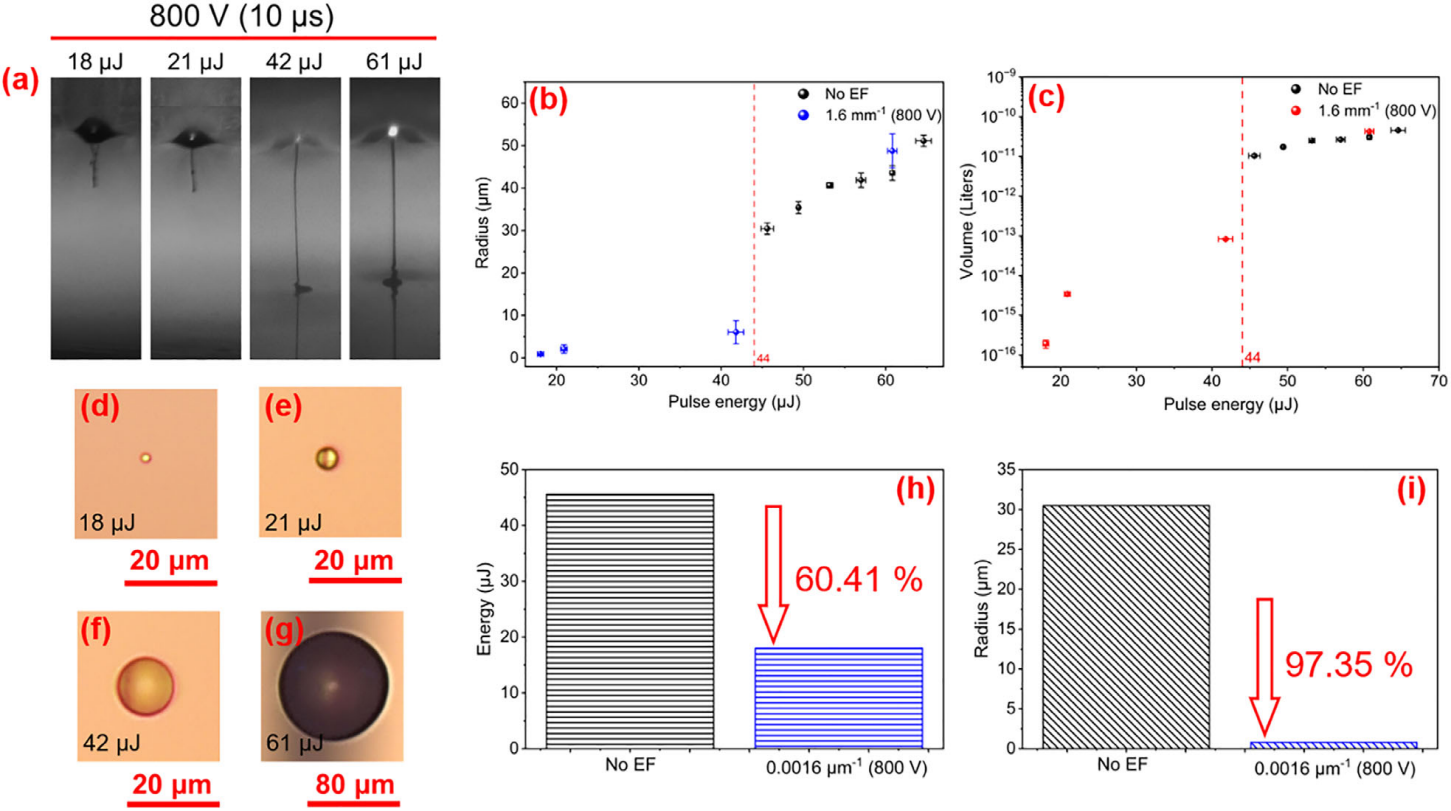
Comparing transfer characteristics with (∼800 V) and without EFs – Donor 1, 170 µm donor thickness. (a) Time resolved imaging of the jet formation at 10 µs and 800 V under various pulse energies. (d–g) transferred droplets from the experiments shown in (a). (b) Radius and (c) calculated volume of the transferred droplets vs laser pulse energy with or without EF. Summary of (h) pulse energy requirement and (i) transfer droplet radius for LIFT with and without EF. Error bars in (b, c) represent the standard deviations in the droplet radius, volume, and the pulse energy.

To further explore the applicability of EHD‐LIFT, additional experiments were conducted using thinner donor layers (∼20 µm). Figure 8 presents time resolved images acquired at 10 µs for thin donors under laser irradiation with and without EFs. The results demonstrate that fine, slender jets can be generated at relatively low EF strengths even under conditions where no jetting occurs in the absence of an EF. The jets obtained with thin donors closely resemble those shown in Figure 8 but are achieved at even lower pulse energies and EF strengths. These findings suggest several potential advantages of EHD‐LIFT, including the possibility of reducing the laser pulse energy required for transfer of energy sensitive materials and providing enhanced control for liquid systems where preparation of uniformly thin donor films is challenging. However, extension to different material systems requires consideration of several factors, including electrochemical effects associated with the applied DC EF, electromigration of suspended particulates such as nanoparticles, and differences in the electronic properties of the donor materials. Therefore, extensive investigation is necessary to find suitable donor material and electrode pair that will have a broad working voltage range before any fluid instabilities or surface phenomena dominate, as demonstrated through this work. Additional discussions on service lifetime and stability of the electrode are made in the Supplementary Information to provide insights on the practical implementation.

**FIGURE 8:**
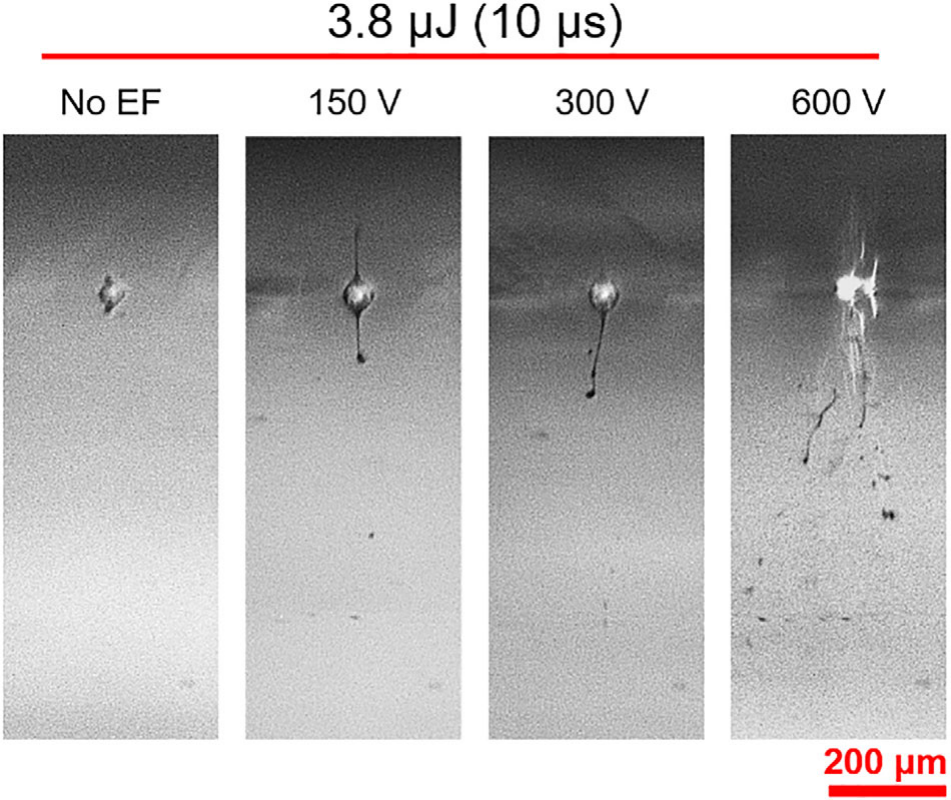
Transfer characteristics of thin donors (∼20 µm) under a laser irradiation of 3.8 µJ at 10 µs with and without EFs.

## 4 Conclusions

This study demonstrates the effects of external EFs during laser‐induced forward transfer of liquid donors. A number of conclusions can be realized:

- Application of DC EFs through interdigitated electrodes leads to local deformations in the donor with alternating concavities and convexities.
- DC EFs produce stable concavities which enable flow focusing in the donor during the LIFT process.
- Conventional LIFT process for the given donor (thicknesses of 170 µm) has a threshold of ∼46 µJ, whereas a threshold of ∼18 µJ is realized with EFs, leading to an energy requirement reduction of 60.41%.
- Minimum transfer radius of ∼30.48 µm could be obtained without EF, whereas a minimum radius of ∼905.5 ± 100 nm is realized with EFs: above a 30‐fold reduction.
- Lower pulse energies (∼3.8 µJ) and EF strengths (150 V) can be utilized to generate small transfer volumes with thinner donors (∼20 µm), similar to that of thicker donors (∼170 µm).

This study experimentally establishes that the applied EF provides an independent means of controlling donor interfacial curvature and that this curvature systematically modifies the resulting jetting dynamics. To support this mechanism, experimental characterizations have been made for the donor curvature as a function of applied voltage and subsequently correlate the curvature with jet length, jet velocity, jet propagation, jet tilting, and transfer characteristics. The observed dependence of jet evolution on donor curvature is also consistent with the previously reported flow focusing mechanism. Future works may include a fully coupled EHD and laser induced bubble/jet flow model that would provide valuable additional insight into the detailed momentum and velocity fields during jet formation. Summarizing, lower donor thickness with low EF strengths are suitable towards optimization of the EF assisted LIFT process. The presented results demonstrate EFs in the LIFT process as an additional degree of freedom to tune jetting characteristics. This tunable parameter may further open up new prospects in LIFT of energy sensitive materials for unprecedented resolution.

## 5 Materials and Methods

A conventional LIFT setup with a UV (343 nm) nanosecond pulsed (∼1 ns) laser (FLARE NX 343‐0.2‐2, Coherent) is used to irradiate a Ti absorption layer (50 nm on 1 mm glass slide) through a 15× objective with 0.31 NA (LMU 15X‐351) to transfer a liquid donor of thickness ∼170 ± 20 µm as shown in Figure 9A. The beam spot diameter at the Ti layer is ∼30 µm. The donor used, is an optical glue (Norland Optical Adhesive 61) with surface tension of 40 dynes/cm, viscosity of ∼0.3–0.4 Pa.s, density of 1231 kg/m^3^ and a dielectric constant of ∼15 [30]. Rheological properties of the donor are checked and reported in Figure S11. The pulse energy is controlled using an optical setup consisting of a quarter wave plate between two linear polarizers. The output beam is split using a beam splitter into 95% to 5% ratio and the 5% beam energy is measured using a photodiode sensor (PD10‐V2‐ROHS from OPHIR). The Ti absorption layer is laser processed to pattern the Ti absorption layer into an interdigitated electrode structure as shown in Figure 9b using a commercial laser workstation (Rofin PowerLine F20, Coherent, with a JENOPTIK JENar FC1800 f‐Theta scan lens, f = 100 mm). The patterned Ti layer is treated to improve liquid donor wetting using a plasma cleaner (Piezo Brush PZ3, module Nearfield). The liquid donor is spread on top of the interdigitated electrode structure using an automated doctor blade coater machine (blade holders 1117/100 mm and 1117/150 mm from Sheen Instruments, on top of an automatic coater TQC Sheen Automatic Film Applicator Compact from Industrial Physics B.V.). The prepared donor is inverted and placed as per Figure 9a. Subsequently, the electric field is applied and controlled using a high voltage DC power supply (Pasco SF‐9586 6 kV power supply, USA) through the +ve and ‐ve electrodes of the Ti absorption layer (see Figure 9b), leading to deformations in the donor layer as illustrated in Figure 9c. This way, LIFT can be conducted with and without EFs by simply turning the voltage source on or off. To image the LIFT transfer process a monochromatic camera (Thorlabs Zelux CS165MU1/M) is used with a 5x objective lens (Nikon Motic Plan Apo ELWD 5x/0.14) and a tube lens (Thorlabs LA 1708A, f = 200 mm) in front of it acting as a simple microscope. The illumination is made through a light emitting diode (LED) (LED Driver Rev. 3_0_1 from Tag Optics), which can be pulsed and triggered using a waveform generator. The laser, motorized stages, camera and the illumination light are all synchronized through a pulse/delay generator together in a way that the camera captures the image of the transfer process as early as 1 µs after the laser is fired. Characterizations are made for the donor layer with or without EF. Confocal microscopes (EVIDENT LEXT OLS5500 Optical Profilometer and Keyence VK‐X3050) are used to image and measure the donor (thickness and deformations) and the transferred material. Additionally, an optical microscope (Zeiss AXIO Imager A1) is utilized to experimentally measure the droplet radius. For droplets that are not perfectly circular, the area is determined using ImageJ processing and an effective radius (*r*) is extracted and used as the radius of the droplet. Subsequently, in order to estimate the droplet volume, the spherical cap approximation is used:

(1) $$V=\frac{1}{3}\;\pi h\left(3r^{2}+h^{2}\right)$$

where *r* is the radius of the drop and *h* is the height of the drop. The height of the drop is experimentally determined using a confocal microscope.

**FIGURE 9:**
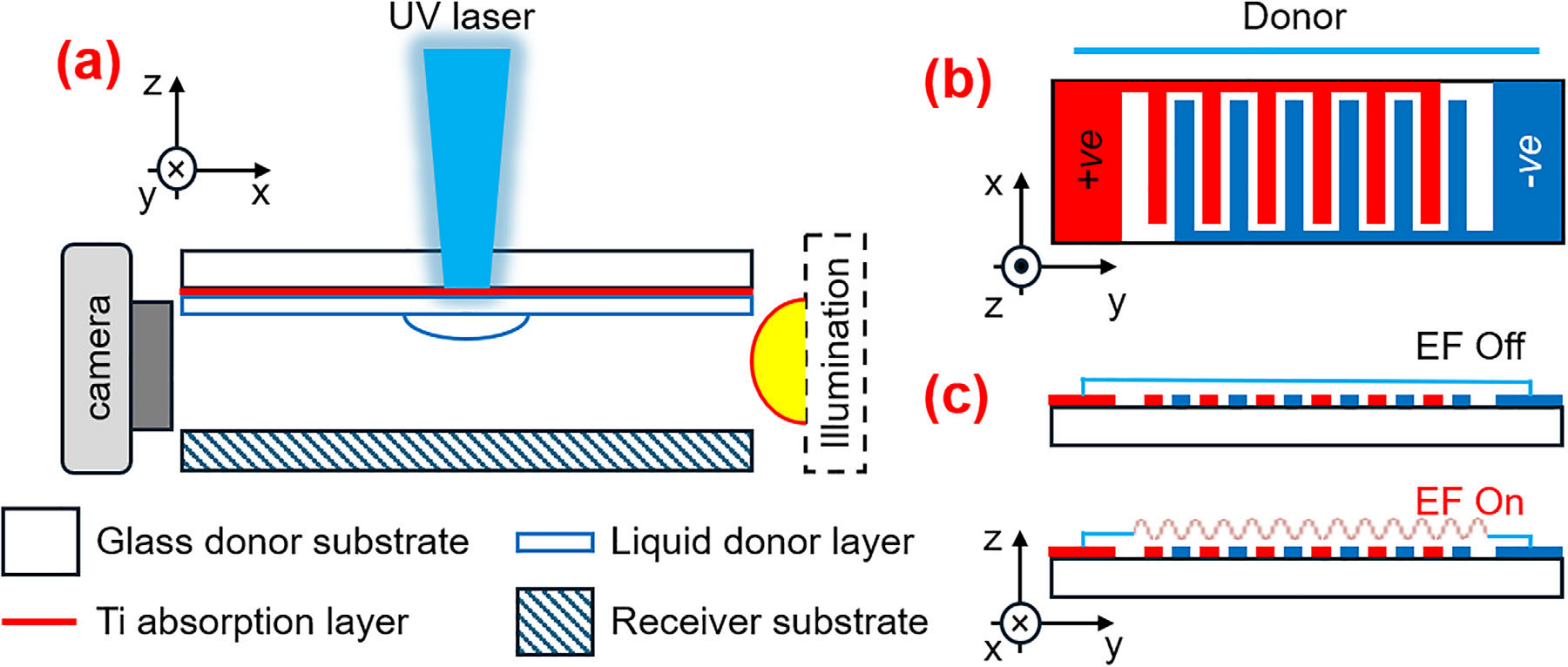
(a) Schematic of the LIFT setup. (b) Schematic of the glass donor substrate with Ti layer, laser scribed into interdigitated electrode configuration. (c) Side view of the donor layer with and without EF.

Amperometric values are taken using a standard multimeter handheld oscilloscope (Tenma 74–8400) connected in series to the EF circuit after the electrodes. EIS measurements were taken using an impedance analyzer (Gamry), considering the donor as the electrochemical system (includes the Ti electrodes imitating the electrodes and the donor liquid as the electrolyte).

## Author Contributions

**Ankit Das**: conceptualization, investigation, writing – original draft, writing – review and editing, visualization, validation, methodology, software, formal analysis, data curation. **Jordi Torres‐durall**: data curation, investigation. **J. Marcos Fernandez‐Pradas**: data curation, investigation, visualization. **Chien‐Fang Ding**: resources. **Martí Duocastella**: resources, supervision, writing – review and editing, funding acquisition, conceptualization. **Craig B Arnold**: resources, supervision, project administration, writing – review and editing, funding acquisition, conceptualization.

## Funding

A.D. and C.B.A. acknowledge financial support from Princeton University. M.D. acknowledges funding from the European Research Council (ERC) under the European Union's Horizon 2020 Research and Innovation Program (grant agreement No. 101002460).

## Conflicts of Interest

The authors declare no conflicts of interest.

## Supporting information




**Supporting File 1**: advs78600‐sup‐0001‐SuppMat.docx.

**Supporting File 2**: advs78600‐sup‐0002‐VideoS1.mov.

## Data Availability

The data that support the findings of this study are available on request from the corresponding author. The data is not publicly available due to privacy or ethical restrictions.
