# Supplementary material for: EHD‐LIFT: Electrohydrodynamic Laser Induced Forward Transfer
Source: Adv Sci (Weinh). 2026 Oct 31:e78600. Online ahead of print. doi: 10.1002/advs.78600 (PMC13723705; doi:10.1002/advs.78600)
Supplement: Supplementary file 1 [file ADVS-9999-e78600-s002.docx]

Supporting Information

EHD-LIFT: Electrohydrodynamic laser induced forward transfer

Ankit Das, Jordi Torres-Durall, J Marcos Fernández-Pradas, Chien-Fang Ding, Martí Duocastella, Craig B Arnold*

**Time resolved imaging of LIFT**

First, we begin analysis of the jetting characteristics of the LIFT process to visually understand the transfer mechanism of the viscous liquid donor (optical adhesive) using time resolved imaging. The LIFT transfer mechanism for the optical adhesive follows a three-step process: (i) bubble formation, (ii) onset of jet formation accompanied by bubble retraction, and (iii) complete bubble collapse followed by jet development. Experimental results validate this mechanism through time resolved imaging as illustrated in **Figure** **S1**. At low pulse energies, bubble formation is observed, which gradually evolves into a small protruding jet. However, owing to insufficient pulse energy, the jet retracts back into the donor without resulting in material transfer **(Figure** **S1(a))**. In contrast, increasing the pulse energy (> 46 μJ) leads to the formation of a larger bubble and subsequent jet formation. A fully developed jet is observed which, in the presence of a receiver substrate would result in successful transfer. However, at the presented pulse energy jet breakup does not occur, and the jet retracts due to the combined effects of the high viscosity and surface tension of the donor liquid as observed at 200 μs in **Figure S1(b)**. Quantitative characterization the jet lengths further confirms that, for lower pulse energies < 46 μJ (insufficient for jetting), the jets retracts before stable propagation can occur **Figure S1(c)**. These results are typical of LIFT of high viscosity inks [1, 2].

**Figure S1.** Transfer mechanism for LIFT of liquid donor irradiated under pulse energies of (a) 45.6 μJ and (b) 60.8 μJ. (c) Jet length evolution for LIFT without EFs with different pulse energies. Note: In (a), larger temporal intervals are used to highlight jet retraction, whereas in (b), shorter intervals are employed to capture the evolution of the bubble and jet dynamics.

Confocal measurements show the deformed liquid donor interface forms a wavy structure with peaks aligned on top of the inter-electrode gaps and the valleys positioned above the electrodes. The radius of curvature (ROC) can be estimated (**Figure S2**) from the experimentally derived curvature (κ) values and is given as per **Equation (S2) and (S1)** respectively. For comparisons with the work of Turkoz et al. [3], the curvature ($\kappa^{'}$) is calculated using the geometrical information of a meniscus, with a meniscus angle of 85.2° in a cylindrical hole of radius (R) 45 μm and 35 μm as per **Equation (S4)**.

$\kappa=\frac{\left| f^{''}(x) \right|}{\left( 1+\left[ f^{'}\left( x \right) \right]^{2} \right)^{\frac{3}{2}}}$	(S1)

$ROC=\frac{1}{\kappa}$	(S2)

$\kappa^{'}=\frac{\cos\theta}{R}$	(S3)

$ROC^{'}=\frac{1}{\kappa^{'}}$	(S4)

**Figure S2.** Confocal experiment based estimations for the radius of curvature of the deformed liquid donor at the ground electrode for different electrode configurations.

For visualization of the EF distributions, a FEM simulation is carried out on COMSOL Multiphysics. The geometry of the setup includes a set of interdigitated electrodes as shown in Fig. 1. Electrostatics module is realized to generate the EF and estimate the field strength inside the donor. The boundary conditions are given through the maxwell’s equations [4]. Results show EF lines diverging out from the live electrode (**Figure S3**) into the ground electrode giving intuition of the local forces that are generated in the liquid donor.

**Figure S3.** FEM based results for applied potential and normalized EF for electrode configuration 1 and a donor layer thickness of 170 μm.

**Figure S4.** Time resolved imaging of transfer characteristics at constant pulse energy and variable EF strength (a) No EF, (b) 500 V, (c) 700 V, (d) 1000 V. Electrode configuration: Donor 1.

**Figure S5.** Time resolved images of jetting behavior under fixed pulse energy with and without EFs (a) No EF, (b) 300 V, (c) 500 V, and (d) 700 V.

**Extended effects under high EF strengths**

Donor 1 consisting of electrode widths of 200 μm and inter-electrode spacing of 100 μm is utilized for most experiments. **Figures** **S6(a,b)** presents jetting dynamics and transfer behavior for different electrode configurations under fixed EF and pulse energy conditions. Changing the electrode geometry and the gaps between them significantly alters the donor interfacial profile and consequently the jetting behavior as visually observed in **Figures S6(a,b)**. This observation can be understood through the electrode configurations, the respective local curvatures and geometry above the electrodes using confocal imaging (**Figure 2(c-e)**).

**Figure S6.** Time resolved imaging of jetting behavior at a pulse energy of 53.2 μJ and 1000 V. (a) Donor 1, (b) Donor 3. (c-f) Confocal results of the donor interfaces above the ground electrode for electrode configurations (c) 1, (d) 2 and (e) 3 (inset: zoomed in image of the local curvature). (f) Jet length evolution for Donors 1 and 3 shown in (a) and (b) respectively.

Under an applied voltage of 1000 V, Donor 1 (electrode width: 200 μm, gap: 100 μm) exhibits a curvature of ~ 2.2 mm^-1^ (**Figure 2(c)**), whereas Donor 2 (electrode width: 400 μm, gap: 200 μm) shows negligible curvature and very low donor thickness at the irradiation site (ground electrode) under the same conditions (**Figure 2(d)**). As a result, no jetting or transfer is observed for Donor 2 due to ablation destruction at 53.2 μJ and 1000 V. Supplemental experiments are conducted with Donor 2 at lower pulse energies and EF strengths **(Figure S7)** to analyze the jetting behavior and avoid the destruction of the donor. Since Donor 2 contains wider electrodes and larger inter electrode gaps than Donors 1 and 3, confocal measurements reveal large peak to valley lengths but correspondingly larger radius of curvature and reduced donor thickness beneath the irradiation site. Under these conditions, lower pulse energies (<30 µJ) and EFs (applied voltage of 700 V) are sufficient to initiate jetting, as experimentally confirmed in **Figure** **S7(a).** While Donor 1 had higher curvature, donor 2 could be jetted at lower energies. **Figure S7(a)** shows the effect of pulse energy, increasing both pulse energy and applied voltage further depletes the donor thickness at the electrode, eventually leaving only a minimal amount of donor material beneath the concavity relative to the deposited laser energy. Under these conditions, explosive ablation-like behavior is observed, as shown in **Figure** **S7(b)** at 1500 V.

**Figure S7.** Time-resolved imaging of jetting dynamics utilizing Donor 2 at (a) constant pulse energy and EF strength (jet evolution with time), and (b) constant pulse energy and time instant (increasing voltage). Electrode configuration: Donor 2

Donor 3 (electrode width: 100 μm, gap: 100 μm) exhibits a spatially intermittent curvature (**Figure 2(e)**). **Figure S6(a,b,c)** also indicates slower jet evolution for Donor 1 compared to Donor 3. This behavior is attributed to both reduced flow focusing, fluid instability and the formation of larger bubbles relative to the local electrode thickness at which the concavity is formed. Additional time resolved experiments for Donor 3, including control conditions and 700 V operation is shown in **Figure S8**. Results are in line with previous results, i.e. application of an EF leads to longer jet formation compared to that of without EF. Overall, these results demonstrate that EF assisted LIFT introduces an additional degree of freedom for tuning jetting dynamics through control of donor interfacial curvature.

**Figure S8.** Time resolved imaging of transfer characteristics at constant pulse energy, with and without EF. Electrode configuration: Donor 3

**Figure S9.** Tilting of jets observed at different locations of the curvature under a voltage of 500 V, laser pulse energy of 45.6 μJ and time stamp of 5 μs. Electrode configuration: Donor 1

While the jet tilt depends on the laser irradiation position on the electrode and the corresponding local donor curvature, time-resolved imaging provides insight into the simultaneous evolution of the jet tilt angle, length, and velocity. **Figure S10(a)** presents a sequence of time-resolved images for a jet generated with the laser focused approximately 40 μm from the electrode center. The images reveals that the jet length, velocity, and tilt angle do not evolve monotonically but instead exhibit three distinct regimes: (i) bubble expansion and collapse, (ii) intermediate jet growth, and (iii) long-jet evolution. Immediately following laser irradiation, the jet length increases rapidly within the first microsecond as the vapor bubble expands. As the bubble collapses, the apparent jet length temporarily decreases before increasing again, marking the onset of the intermediate jet regime (**Figure S10(b)**). During this stage, the jet length increases approximately linearly until a transition occurs at around 10 μs, after which the jet enters the long-jet regime characterized by continued elongation.

**Figure S10.** (a) Time-resolved imaging of the jet when laser is irradiated at electrode location of ~40 μm. Evolution of (b) jet length, (c) jet velocity and (d) jet angle as per experimental conditions in (a). Width of the electrode: 200 μm; Gap: 100 μm.

The evolution of the jet velocity, shown in **Figure S10(c)**, closely follows these three regimes. An initial decrease in velocity accompanies bubble collapse, as the protruding jet advances more slowly than the retracting bubble interface. This is followed by a nearly linear increase in velocity during the intermediate regime, indicating approximately constant acceleration. As the jet transitions into the long-jet regime, the velocity gradually decreases while the jet continues to elongate. The tilt angle exhibits a similarly distinct three stage evolution (**Figure S10(d)**). During bubble collapse, the jet progressively aligns with the local surface normal, resulting in an increase in tilt angle. In the intermediate regime, the tilt angle remains nearly constant despite continued jet growth. However, once the jet enters the long jet regime, the tilt angle gradually decreases until the jet becomes nearly vertical. This reduction in tilt may arise from two contributing mechanisms: (i) the increasing gravitational loading of the elongated jet relative to the driving jetting force, and (ii) the formation of a fluid bridge between the donor and receiver substrates, which constrains further lateral deflection.

**Figure S11.** Rheological properties of the donor.

Ohnesorge number (Oh) gives the ratio of the viscous forces to the inertial and the surface tension forces and is given as per Eq. (S5). Physically, in jetting, Oh characterizes the jet stability and droplet breakup, independent of velocity. When Oh > 0.1 stable jets are produced. Calculations show that for the utilized donor the Oh is > 0.1 thereby leading to jetting which is observed as per the results obtained in this work.

$Oh=\frac{\mu_{0}}{\sqrt{\rho\sigma H}}=\frac{0.3}{\sqrt{1231\times0.04\times170\times{10}^{-6}}}\cong3.278$	(S5)

Where, $\mu_{0}$ is the zero shear viscosity, $\rho$ is the density, $\sigma$ is the surface tension, $H$ is the donor thickness.

**Array printing results**

To measure the standard deviations in the dimensions of the transferred material, droplet arrays are made on standard glass slides (microscope slides) as shown in Figure. S12. Table S1 shows some statistical evaluations of the array printing. The methodology for estimation of radius and volume is given in the materials and methods section in the manuscript. Circularity is determined through standard ImageJ processing tool. Repeatability is determined through the following formula:

$Repeatability=\frac{successful transfers}{total number of transfer attempts}\times100\%$,	(S6)

where total number of transfer attempts is fixed at 12 for array printing demonstrations.

**Table S1**.

| **Experimental parameters** | | **Radius**  **(μm)** | **Volume**  **(L)** | **Circularity** | **Repeatability (%)** |
| --- | --- | --- | --- | --- | --- |
| **Pulse energy (μJ)** | **Voltage (V)** |  |  |  |  |
| 45.6 ± 0.76 | No EF | 30.48 ± 1.31 | 1.04 ×10^-11^± 9.02×10^-13^ | 0.935 ± 0.055 | ~83.3 |
| 49.4 ± 0.19 |  | 35.42 ±1.39 | 1.73 ×10^-11^± 1.36×10^-12^ | 0.916 ± 0.02 | ~91.6 |
| 53.2 ± 0.38 |  | 40.64 ±0.61 | 2.52 ×10^-11^± 7.50×10^-13^ | 0.88± 0.03 | ~100 |
| 57 ± 0.57 |  | 41.86 ±1.74 | 2.67 ×10^-11^± 2.23×10^-12^ | 0.900 ± 0.05 | ~100 |
| 60.8 ± 0.28 |  | 43.56 ±1.73 | 3.05 ×10^-11^± 2.44×10^-12^ | 0.899 ± 0.05 | ~100 |
| 64.6 ± 0.95 |  | 51.11 ±1.31 | 4.54 ×10^-11^± 2.44×10^-12^ | 0.892 ± 0.08 | ~100 |
| 18.05 ± 0.38 | 800 V  (Donor 1) | 0.9055 ± 0.1 | 1.928×10^-16^ ± 4.506×10^-17^ | 0.985 ± 0.015 | ~100 |
| 20.9 ± 0.38 |  | 2.103 ± 1 | 3.427×10^-16^ ± 2.232×10^-16^ | 0.980 ± 0.01 | ~100 |
| 41.8 ± 0.95 |  | 6.093 ± 2.74 | 8.334×10^-14^ ± 2.443×10^-15^ | 0.981 ± 0.009 | ~100 |
| 60.8 ± 0.57 |  | 48.748 ± 4 | 4.267×10^-11^ ± 2.327×10^-12^ | 0.954 ± 0.02 | ~100 |

**Figure S12.** Printing arrays of drops with and without EFs under different pulse energies.

**Electrode stability and operational lifetime**

In the present EHD-LIFT configuration, the patterned Ti layer serves a dual purpose as both the laser absorbing layer and the interdigitated electrode for generating the EF for donor curvature. Therefore, successful transfer requires irradiation at locations where the Ti layer remains intact. Since the laser irradiation is localized, multiple transfers can be performed at different, previously unirradiated locations on the same electrode substrate. Considering this, pattern/array transfers are made as shown in **Figure S12**. Within the stable operating regime investigated here (≤1000 V), the electrode configuration remained suitable for repeated processing, and the practical operational lifetime is primarily associated with the available unprocessed Ti area rather than the number of laser shots. To further analyze electrode degradation, additional transfers above approximately 1000 V were made. At this voltage, unstable donor behavior is obtained, and is therefore unsuitable for reproducible EHD-LIFT. However, the same electrode substrate remained functional when the applied voltage was subsequently reduced to ≤1000 V. This indicates that, under the conditions investigated, exposure to the higher voltage regime did not result in irreversible electrode degradation that prevented subsequent operation within the stable regime. Thus, the available Ti absorbing/electrode area represents a more immediate practical limitation than electrode lifetime under the conditions studied.

**References**

1.	Marti Jerez, E., et al., *Substrate Reshaping for Optically Tuned Liquid‐Printed Microlenses Beyond Their Wetting Properties.* Advanced Materials Technologies, 2023. **8**(19): p. 2300564.

2.	Martí‐Jerez, E., et al., *3D Shape Control of Printed Micro‐Electrodes with Substrate Reshaping.* Advanced Materials Technologies, 2025. **10**(7): p. 2401641.

3.	Turkoz, E., et al., *Subthreshold laser jetting via flow-focusing in laser-induced forward transfer.* Physical Review Fluids, 2018. **3**(8): p. 082201.

4.	Maxwell, J.C., *A treatise on electricity and magnetism*. Vol. 1. 1873: Clarendon press.
